# Imported malaria in the UK, 2005 to 2016: Estimates from primary care electronic health records

**DOI:** 10.1371/journal.pone.0210040

**Published:** 2018-12-31

**Authors:** Hamad Bastaki, Louise Marston, Jackie Cassell, Greta Rait

**Affiliations:** 1 Research Department of Primary Care and Population Health, University College London, London, United Kingdom; 2 Division of Primary Care and Public Health, Brighton and Sussex Medical School, University of Brighton, Brighton, United Kingdom; University of Oxford, UNITED KINGDOM

## Abstract

**Objective:**

To investigate trends in the incidence of imported malaria in the UK between 2005 and 2016.

**Design:**

Analysis of longitudinal electronic health records (EHRs) in The Health Improvement Network (THIN) primary care database.

**Setting:**

UK primary care

**Participants:**

In total, we examined 12,349,003 individuals aged 0 to 99 years.

**Outcome measure:**

The rate of malaria recordings in THIN was calculated per year between 2005 and 2016. Rate ratios exploring differences by age, sex, location of general practice, socioeconomic status and ethnicity were estimated using multivariable Poisson regression.

**Results:**

A total of 1,474 individuals with a first diagnosis of malaria were identified in THIN between 2005 and 2016. The incidence of recorded malaria followed a decreasing trend dropping from a rate of 3.33 in 2005 to 1.36 cases per 100,000 person years at risk in 2016. Multivariable Poisson regression showed that adults of working age (20 to 69 years), men, those registered with a general practice in London, higher social deprivation and non-white ethnicity were associated with higher rates of malaria recordings.

**Conclusion:**

There has been a decrease in the number of malaria recordings in UK primary care over the past decade. This decrease exceeds the rate of decline reported in national surveillance data; however there are similar associations with age, sex and deprivation. Improved geographic information on the distribution of cases and the potential for automation of case identification suggests that EHRs could provide a complementary role for investigating malaria trends over time.

## Introduction

Globally, malaria affected 216 million individuals in 2016 and resulted in 445,000 deaths [1]. Amongst non-endemic countries, the UK has one of the highest numbers of imported malaria in Europe with around 1500 cases a year [2]. Despite a decreasing trend in the overall global number of cases, it remains the most common imported infection in returning ill travellers in Europe [3].

In the UK, malaria is a notifiable disease and information on trends over time is usually obtained from passive surveillance data on imported cases from the Malaria Reference Laboratory (MRL), supplemented more recently with cases reported to the Public Health England (PHE) case management database (HPzone) [2, 4]. Reports on cases are completed by clinicians and laboratory staff with information on patient demographics, reason for travel, area and duration of travel, and chemoprophylaxis use [5]. This surveillance method is associated with underreporting since not all cases who seek healthcare are notified. A capture recapture study estimated that only 56% of cases were captured by the MRL surveillance system [6], with similar levels of underreporting in other European notification based surveillance systems [7–9].

Use of electronic health records (EHRs) may provide a complementary method of exploring trends in malaria diagnoses over time. Although they have limitations, EHRs have been widely used for observational research and several studies have used them for exploring trends in the incidence of illnesses over time [10–13]. Data collection at the time of recording a malaria diagnosis in primary care can result in the inclusion of unreported cases in the analysis as it forgoes the added step of notification. Furthermore, it can provide insight into the number of cases seen in this setting.

Our aims were to investigate the incidence of imported malaria in the UK between 2005 and 2016 using The Health Improvement Network (THIN) Primary care database; to explore the variation in incidence by age, sex, UK region, socioeconomic status and ethnicity; and to compare the characteristics of cases identified in THIN to those identified through passive surveillance.

## Methods

### Data source

Relevant data were extracted from THIN. THIN contains anonymised EHRs of around 12 million patients attending 693 general practices participating in the network [14]. It has coverage of around 6% of the UK population and has been shown to be broadly representative of the UK population in terms of demographics and prevalence of major conditions [15]. Data in THIN are collected routinely from consultations in general practice and consist of information on individual patient characteristics, medical information such as symptoms and diagnoses of disease, investigations ordered and medication prescribed. Data are coded using Read codes, a standard vocabulary list clinician’s use to record medical information; and drug codes, based on the British National Formulary classifications for medication.

### Study population

General practices that contributed data to THIN between January 2005 and December 2016 were used for this study. The quality of the data included was assessed using the acceptable computer usage (ACU) dates [16] and the acceptable mortality recording (AMR) dates [17]. The ACU date refers to the date when general practices use their computer systems adequately for recording clinical data. In THIN, this was defined as the date where at least one medical record, one additional health data (AHD) record and two therapy records are consistently recorded per patient per year[16]. The AMR date refers to the date from which the mortality recordings of a general practice are deemed complete. In THIN, this was defined as the date the mortality recordings within the general practice was similar to what the expected mortality would be in the general population using the Office for National Statistics data, taking into account the characteristics of the patients within that practice [17]. Practices were included after the latter of the ACU and AMR date. Additionally, information on postcode linked socioeconomic indices (Townsend Score) was not available for 17 practices and they were excluded from the analysis.

### Participants

All individuals aged 0–99 years that were registered with a general practice contributing data between 2005 and 2016 were included in the analysis.

#### Case definition

**Fig 1:**
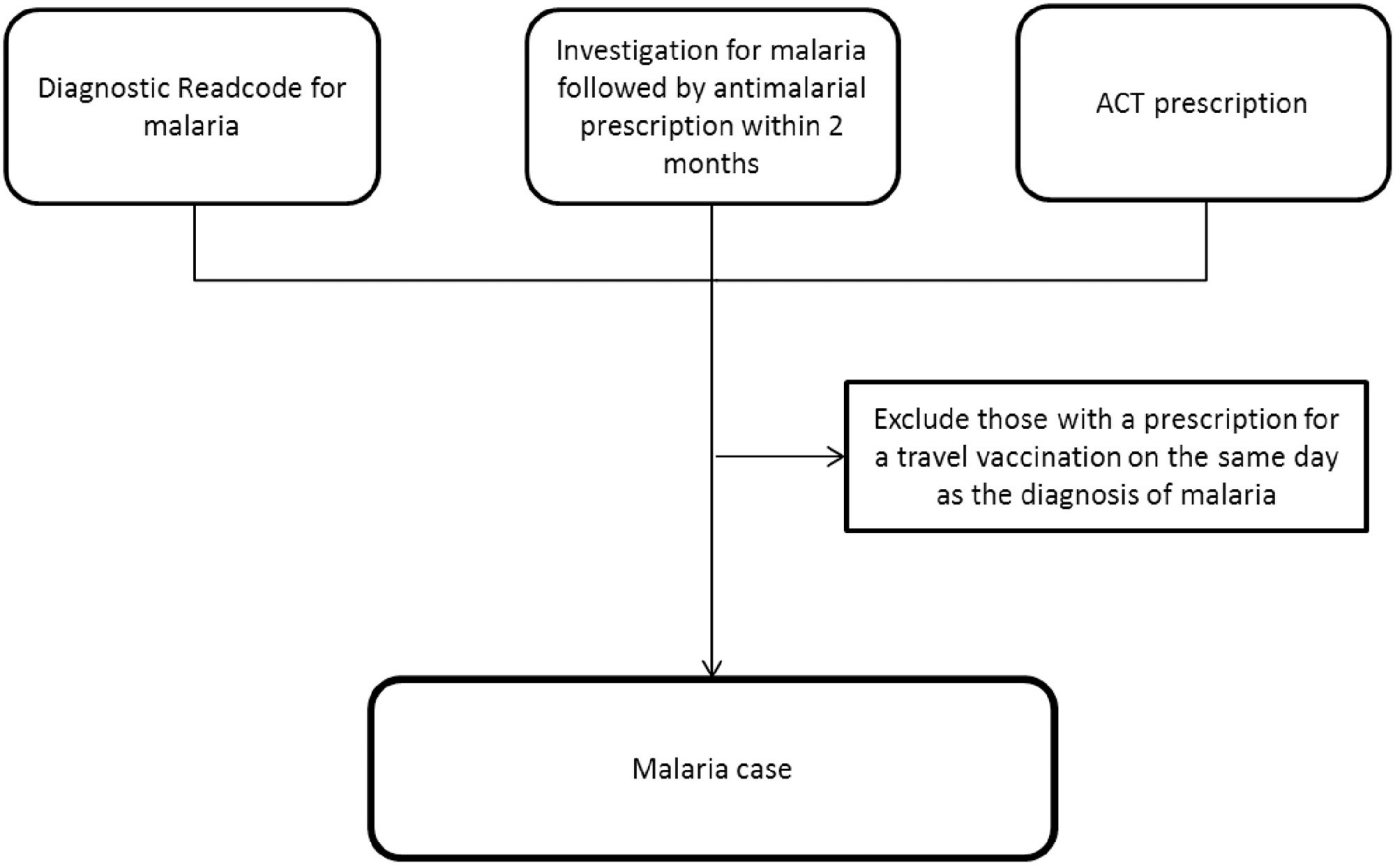
An algorithm summarising how malaria cases were identified within THIN database. THIN: The Health Improvement Network, ACT: Artemesinin-based combination therapy. To minimise the effect of miscoding a diagnosis of malaria for a pre-travel malaria advice consultation, those with a prescription for a travel vaccination issued on the same day as a malaria recording were excluded (S1 Table).

Cases of malaria in primary care may be indicated by diagnostic codes, or a combination of investigation and treatment codes. An algorithm was developed to identify individuals diagnosed with malaria within THIN based on having any of the following records (Fig 1):

- **A diagnostic Read code for malaria**:
  A Read code list indicating a diagnosis of malaria was developed (S1 Table). Those with a malaria diagnostic Read code within their medical records were considered to have a diagnosis of malaria. The date that malaria was first recorded was considered the index date of diagnosis.
- **A code for malaria investigation followed by treatment for malaria within 60 days**:
  In order to capture potential cases that did not have a diagnostic Read code for malaria, those with an AHD code or Read code for malaria specific investigations were identified (S1 Table). In records where a laboratory test is ordered with no result reported in THIN, it was assumed that the individual had a diagnosis of malaria if an antimalarial was prescribed within two months after the date the test was ordered. The antimalarials prescribed were identified using a drug code list developed based on chapter 5.4.1 of the British National Formulary (BNF) (S1 Table) [18]. Although some of these medications can be used for prophylaxis, the preceding indication for ordering the malaria specific investigation makes this unlikely. The date the antimalarial was prescribed was considered the index date of diagnosis in those who were investigated for malaria.
- **A prescription for artemesinin-based combination therapy (ACT)**.
  Artemesinin-based combination therapy is the recommended treatment for those with *P*. *falciparum* infection and for non-falciparum malaria if the area of infection is known to have chloroquine resistance [19]. The drug codes in THIN were searched to identify all ACTs recorded in the database and only artemether–lumefantrine was identified. Artemether–lumefantrine is the drug of choice for treating uncomplicated *P*. *falciparum* and is licensed solely for the treatment of malaria [18]. It was assumed that the individual had a diagnosis of malaria if they received a prescription for artemether-lumefantrine. The date this was prescribed was considered to be the index date of diagnosis.

### Analysis

The overall crude incidence of malaria was estimated per 100,000 person years at risk (PYAR). This was determined by totalling the number of patients with a first recording of malaria between 2005 and 2016, and then dividing this number by the total person years of follow-up for all patient records for this period. The start date used to calculate the total PYAR for each participant was the latest of:

- The date the individual registered with their current general practice
- The date the practice reached AMR
- The date the practice reached ACU
- The 1^st^ of January 2005

The end date used to calculate the total PYAR for each participant was the earliest of:

- The date the individual transferred to a different practice to the one included in the analysis.
- The date of death recorded in the practice.
- The last date the practice contributed data to THIN.
- The index date of diagnosis in those who have malaria
- The 31^st^ of December 2016

Crude incidence rates by calendar year, age group (<10, 10–19, 20–29, 30–39, 40–49, 50–59, 60–69, 70–79, 80–89 and >90 years), sex (male and female), level of social deprivation (Townsend Score; a measure which incorporates unemployment, car ownership, home ownership and household overcrowding to calculate area-level deprivation. The score ranges from 1 to 5 with 1 being the least deprived and 5 indicating the greatest degree of deprivation) region (based on former strategic health authority location; London, East Midlands, East of England, North East, North West, Northern Ireland, Scotland, South Central, South East Coast, South West, Wales, West Midlands and Yorkshire and Humber) and ethnicity (Grouped into the 2001 UK census 5 category classification: White, Black, Asian, Other and Mixed) were also estimated by restricting the person years of follow-up to the respective category in question.

Multivariable Poisson regression analysis was carried out to explore the change in incidence by calendar year, age group, sex, region and Townsend score mutually adjusting for the other variables included in this model. Ethnicity was not included in the model as the data quality was poor with missing data in 40% of cases. To fit the Poisson model to calculate a rate ratio, the coefficients were exponentiated with person-time specified as the exposure.

Additionally a sensitivity analysis was conducted to take into account multiple episodes of malaria in the same individual. It was considered an individual had an additional episode of malaria if they had a diagnostic Read code for malaria at least three months after the previous recording. In these individuals, the start date used to calculate PYAR commenced three months after the date of the previous event as they were not considered to be at risk during that three month period.

All statistical analyses were carried out using Stata version 14 [20].

### Comparison with national surveillance data

Data on the number of malaria cases imported to the United Kingdom from 2005 to 2016 by UK region was obtained from the PHE Travel and Migrant section. In the absence of an equivalent denominator for PYAR to estimate incidence for PHE data, we compared the number of cases by year and UK region as a proportion of the total number of cases for each dataset from 2005 to 2016 to describe the variation in the number of cases identified over time, and their geographical spread in THIN compared to those notified to PHE.

### Ethics

THIN data collection has been approved by the South East NHS Multicentre Research Ethics Committee. Scientific approval for this study was obtained from the IMS Heath Scientific review committee in 2016 (ref: SRC 16THIN056).

## Results

**Fig 2:**
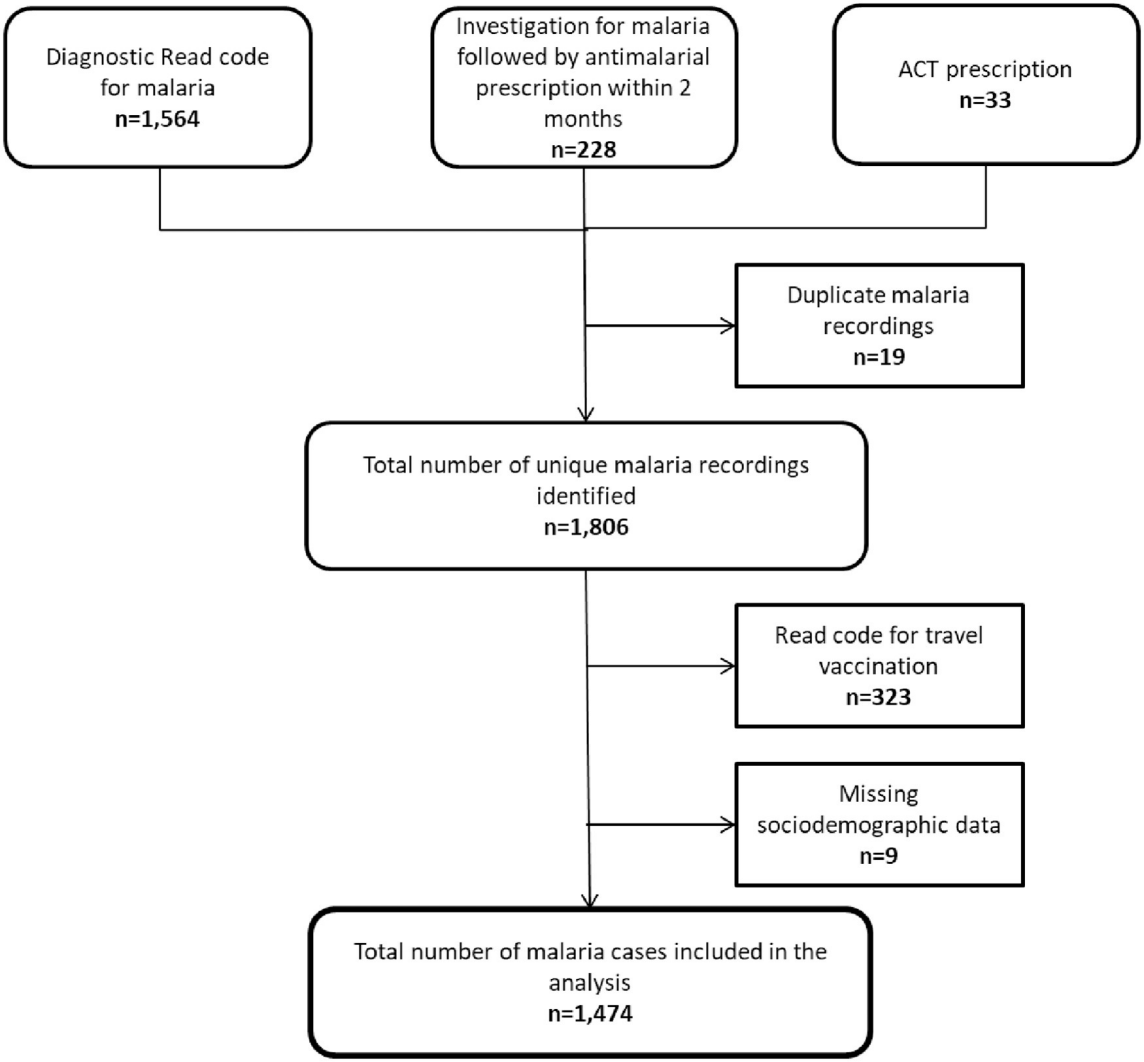
Number of cases identified in THIN from 2005 to 2016. THIN: The Health Improvement Network, ACT: Artemesinin-based combination therapy.

A total of 1,806 individuals with a first diagnosis of malaria were identified in THIN between 2005 and 2016, with 1,474 cases included in the final analysis after excluding those with a code for a travel vaccination on the index date of diagnosis or were missing sociodemographic data. A summary of the number of cases identified at each stage of the algorithm is shown in (Fig 2).

**Fig 3:**
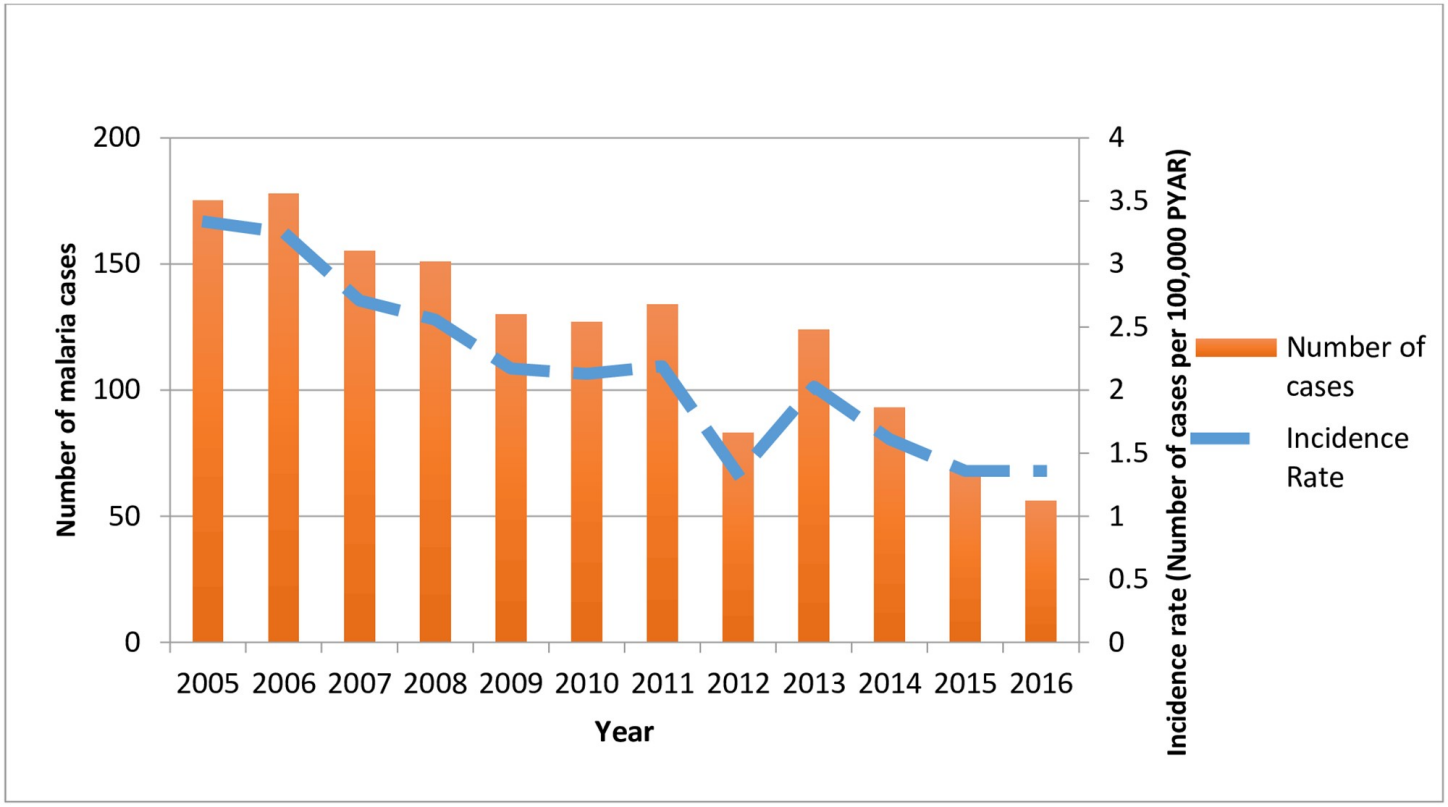
Malaria incidence in THIN, 2005 to 2016. THIN: The Health Improvement Network, PYAR: Person Years at Risk.

The incidence of recorded malaria followed a decreasing trend dropping from a rate of 3.33 in 2005 to 1.36 cases per 100,000 person years at risk in 2016 (Fig 3). The incidence was higher in those aged 20 to 69 compared to those less than 20 years or more than 70 years of age (Table 1). Men experienced a higher rate of recorded malaria than women (IRR: 0.72 (95% CI 0.65–0.80), incidence per 100,000 PYAR; men 2.57, women 1.81). General Practices in London and the East of England had the highest incidence (6.15 and 2.51 per 100,000 PYAR respectively), while practices in Northern Ireland were 89% less likely to have a recording of malaria compared to London (IRR: 0.11 (95% CI 0.07–0.18), incidence per 100,000 PYAR: 0.63) (Table 1, S1 Fig). Greater deprivation was associated with a higher recording of malaria compared to those who were less deprived (IRR: 1.86 (95% CI 1.54–2.25), incidence per 100,000 PYAR; most deprived: 3.29, least deprived: 1.48).

**Table 1:** Incidence of malaria recordings in THIN by calendar year, age, sex, region, Townsend score and ethnicity.

	Number of cases	PYAR (100,000)	Incidence	Unadjusted IRR (95% CI)	Adjusted† IRR (95% CI)
**Year**					
2005	175	52.50	3.33	Baseline	
2006	178	54.81	3.25	0.97 (0.79–1.20)	0.97 (0.79–1.19)
2007	155	57.14	2.71	0.81 (0.66–1.01)	0.81 (0.65–1.01)
2008	151	58.95	2.56	0.77 (0.62–0.96)	0.77 (0.62–0.95)
2009	130	59.80	2.17	0.65 (0.52–0.82)	0.65 (0.51–0.81)
2010	127	59.59	2.13	0.64 (0.51–0.80)	0.62 (0.50–0.78)
2011	134	61.20	2.19	0.66 (0.52–0.82)	0.64 (0.51–0.80)
2012	83	62.42	1.33	0.40 (0.31–0.52)	0.39 (0.30–0.51)
2013	124	61.10	2.03	0.61 (0.48–0.77)	0.59 (0.47–0.74)
2014	93	57.68	1.61	0.48 (0.38–0.62)	0.48 (0.38–0.62)
2015	68	49.97	1.36	0.41 (0.31–0.54)	0.43 (0.33–0.57)
2016	56	41.27	1.36	0.41 (0.30–0.55)	0.44 (0.32–0.60)
**Total**	1,474	676.43	2.18		
**Age**					
Less than 10 years	79	70.44	1.12	Baseline	
10 to 19 years	104	73.08	1.42	1.27 (0.95–1.70)	1.32 (0.98–1.77)
20 to 29 years	249	87.46	2.85	2.54 (1.97–3.27)	2.50 (1.94–3.22)
30 to 39 years	323	96.89	3.33	2.97 (2.32–3.80)	2.81 (2.20–3.60)
40 to 49 years	326	101.02	3.23	2.88 (2.25–3.68)	2.92 (2.28–3.74)
50 to 59 years	213	84.06	2.53	2.26 (1.75–2.92)	2.43 (1.88–3.15)
60 to 69 years	120	71.45	1.68	1.50 (1.13–1.99)	1.69 (1.27–2.25)
70 to 79 years	46	50.66	0.91	0.81 (0.56–1.16)	0.91 (0.64–1.31)
80 to 89 years	9	30.53	0.29	0.26 (0.13–0.52)	0.30 (0.15–0.60)
90 years and older	5	10.85	0.46	0.41 (0.17–1.01)	0.51 (0.21–1.25)
**Sex**					
Male	846	329.05	2.57	Baseline	
Female	628	347.37	1.81	0.70 (0.63–0.78)	0.72 (0.65–0.80)
**Region**					
London	474	77.10	6.15	Baseline	
East Midlands	25	13.74	1.82	0.30 (0.20–0.44)	0.29 (0.20–0.44)
East of England	95	37.80	2.51	0.41 (0.33–0.51)	0.45 (0.36–0.56)
North East	27	13.62	1.98	0.32 (0.22–0.48)	0.32 (0.22–0.48)
North West	80	59.40	1.35	0.22 (0.17–0.28)	0.24 (0.19–0.30)
Northern Ireland	17	27.14	0.63	0.10 (0.06–0.17)	0.11 (0.07–0.18)
Scotland	164	97.51	1.68	0.27 (0.23–0.33)	0.30 (0.25–0.36)
South Central	169	79.79	2.12	0.34 (0.29–0.41)	0.41 (0.34–0.49)
South East Coast	140	74.18	1.89	0.31 (0.25–0.37)	0.37 (0.31–0.45)
South West	73	60.50	1.21	0.20 (0.15–0.25)	0.23 (0.18–0.29)
Wales	71	68.32	1.04	0.17 (0.13–0.22)	0.20 (0.15–0.25)
West Midlands	126	54.58	2.31	0.38 (0.31–0.46)	0.42 (0.34–0.51)
Yorkshire & Humber	13	12.75	1.02	0.17 (0.10–0.29)	0.17 (0.10–0.29)
**Townsend Score**					
(Least Deprived) 1	218	147.02	1.48	Baseline	
2	204	132.21	1.54	1.04 (0.86–1.26)	1.03 (0.85–1.25)
3	290	127.94	2.27	1.53 (1.28–1.82)	1.39 (1.16–1.66)
4	344	111.25	3.09	2.09 (1.76–2.47)	1.82 (1.53–2.16)
(Most deprived) 5	248	75.40	3.29	2.22 (1.85–2.66)	1.86 (1.54–2.25)
Missing	170	82.61	2.06	1.39 (1.14–1.70)	1.15 (0.94–1.42)
**Ethnicity**					
Black	432	7.70	56.14	-	-
White	312	220.00	1.40	-	-
Asian	114	13.00	8.86	-	-
Other	13	4.60	2.83	-	-
Mixed	20	2.40	8.51	-	-
Missing	592	430.00	1.38	-	-
**Total**	1,483ⁱ	677.70	2.19	-	-

THIN, The Health Improvement Network; PYAR, Person Years at Risk; CI, Confidence Interval; IRR, Incidence rate ratio.

^†^IRR adjusted for year, age, sex, region and Townsend score. IRR was not adjusted for ethnicity due to a large amount of missing data (40% missing).

^ⁱ^The total includes the 9 cases with missing demographic information.

Ethnicity was poorly recorded in THIN with missing data on 40% of the cases included in the analysis (Table 1). Amongst those with a recording of ethnicity, those who identify as black had the highest incidence while those who identified as white had the lowest (56.14 and 1.40 per 100,000 PYAR respectively). Those with unrecorded ethnicity data had a similar rate as those who identified as white (1.38 vs 1.40 per 100,000 PYAR).

The sensitivity analysis looking at multiple episodes within the same individual identified nine individuals with two episodes and one individual with more than two episodes of recorded malaria within the study period. Including these episodes in the analysis had no significant effect on the results.

### Comparison with national surveillance data

**Fig 4:**
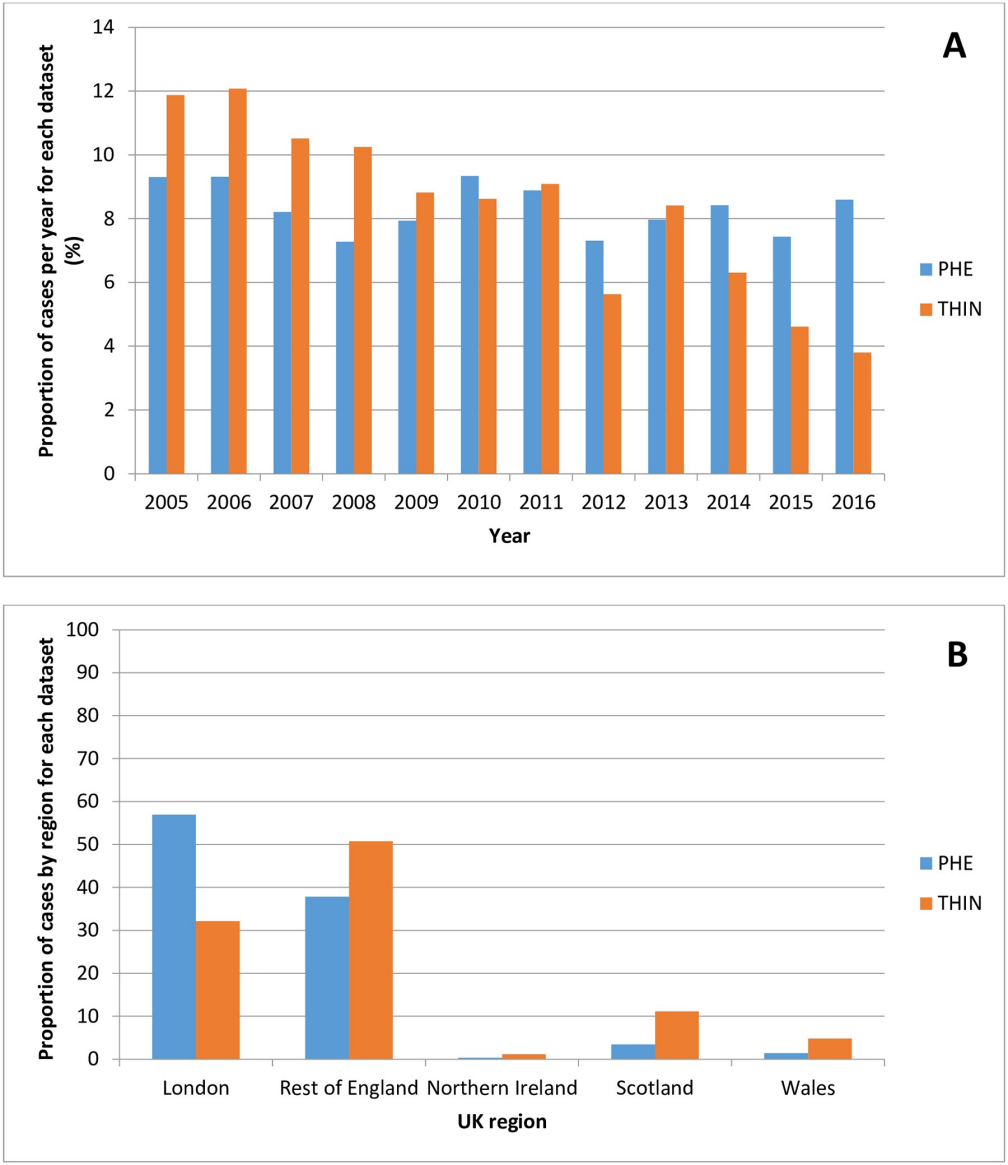
Comparison of the proportion of total malaria cases identified by PHE and THIN from 2005 to 2016. (A) Per year, and (B) by UK region.

There were a total of 18,833 cases of malaria identified by PHE through the MRL and HPZone between 2005 and 2016. Comparing the proportion of cases identified by each dataset per year, there was a proportionately larger number of cases identified in THIN between 2005 and 2009 compared to PHE and a proportionately smaller number of cases in THIN for more recent years (2014 to 2016) (Fig 4A). In terms of region, there was a larger representation of cases from London in PHE (57%) compared to THIN (32%) (Fig 4B).

## Discussion

This study showed that the incidence of malaria recordings in primary care significantly decreased between 2005 and 2016. Adults of working age were three times as likely to have a diagnosis of malaria compared to children and older adults. Men were 28% more likely to have a diagnosis of malaria compared to women. London had the highest incidence of malaria in the UK and those who were most deprived were 86% more likely to be diagnosed with malaria compared to those who were least deprived.

Findings of this study were similar to other data from the UK [2, 21], however the rate of decline in the number of cases was more pronounced compared to data from the national review for malaria, where annual case numbers have remained stable over the last 10 years [2]. The lack of a denominator in our comparison of THIN and PHE data meant that variations over time could either reflect changes in the number of cases identified; or changes in the number of individuals where the cases derived from. Therefore the more pronounced decline in THIN could partly be due to the improvement in data quality with regards to the completeness of PHE data. Since 2013, cases reported to the PHE case management database (HPZone) supplemented the cases identified in the MRL dataset. In addition, the number of cases identified in THIN may be underestimated for 2016 since not all practices contributed data for that year, further contributing to the declining trend. Despite this, it is likely that our findings reflect a true decline in the number of cases since 2005. Decreasing global malaria incidence [1], decreased transmission of malaria in West Africa, where most cases in the UK are acquired and changing chemoprophylaxis usage have been suggested as possible factors contributing to the decline [22, 23].

Similar to data from national and European surveillance [2, 24], men were more likely to present with malaria than women. Previous studies have suggested that gender differences in attitudes towards seeking pre-travel health advice [25], poorer adherence to malaria chemoprophylaxis [26], increased risk taking behaviour and travel to more remote areas where the risk of contracting malaria is higher may account for the male predominance in the number of malaria cases [27]. On the other hand, other studies have shown no gender differences in chemoprophylaxis uptake and adherence [28] and suggest that host factor differences resulting in increased attractiveness to mosquitoes may be responsible for the higher rate of malaria in men [26]. Although both, behavioural and biological host factors may be contributory, the main reason for the difference is likely due to the number of individuals who travel as between 2014 and 2016, 17% more men travelled to malaria endemic areas from the UK than women [29]. Similarly, age differences in malaria rates may also reflect UK travel patterns [29].

Although we were unable to adjust for ethnicity in our analysis due to a large amount of missing data, the trends in the rates of recordings where data were available was comparable with other UK data, showing higher rates in ethnic minorities (Black, mixed, Asian and other, respectively) compared to those who were White[2, 21]. Additionally, those with missing ethnicity data had a similar rate of malaria recording in THIN as those who were white suggesting that the majority of those with missing data were white. Furthermore, those diagnosed with malaria could be more likely to have their ethnicity recorded compared to those who are not, resulting in an inflated value for the incidence rate amongst ethnic minorities. Also, given that ethnic minority populations are more likely to be found in London and are more likely to experience deprivation [30, 31], ethnicity may account for the higher incidence of malaria in London and in those who were most deprived.

Use of EHRs for malaria research could provide a complementary role to data obtained through passive surveillance. The characteristics of each data source and their respective strengths and limitations are summarised in (Table 2). For THIN, the large number of individuals included in the analysis allowed us to accurately compare primary care incidence estimates by age, sex, gender, region, socioeconomic status and calendar year. Additionally, the use of routinely collected prospective data to estimate incidence potentially captured cases not reported in surveillance based incidence estimates, since it relies on clinicians recording a diagnosis of malaria regardless of whether it had been notified to Public Health England or MRL. It also resulted in a more accurate estimation of the geographic distribution of cases since the centralised reporting site for MRL make it less sensitive to cases resident outside of London [6]. On the other hand, relying on clinician recording of the diagnosis also meant that it was difficult to look at the incidence of malaria by parasite species since the species of malaria were rarely recorded. Moreover, although our algorithm captured cases without a diagnostic code for malaria by looking at other variables such as treatment and investigations and excluded those with a code for a travel related vaccination, there still remains a risk of some misclassification if a diagnosis of malaria was not considered. Furthermore, our use of GP records for the analysis of incidence meant that our findings are restricted to those registered with a general practitioner. Using an additional data source from secondary care (Hospital Episode Statistics, HES) could have resulted in additional information on parasite species and identified further cases not recorded in THIN [32]. However, the limited number of practices in THIN with linkage to HES (23% of practices) and the lack of HES-linked data for practices outside England would have restricted the number of individuals included in the study [33]. Despite this, future research on the subset of individuals with HES linked data could provide insight into the levels of recording in both settings. Finally, the number of malaria recordings is not a true reflection of incidence since only those who travel are at risk of contracting malaria, however, in the absence of reliable data regarding travel in THIN, including all individuals when looking at the rate of malaria recordings allows us to explore trends over time and compare with surveillance data which also uses population data [2, 24].

**Table 2:** summarising the characteristics, strengths and limitations of using PHE data and primary care EHR (THIN) data.

	PHE	THIN
**Data source**	Passive surveillance through MRL (supplemented by HPZone since 2013) [2]	Primary care electronic health records
**Case definition for malaria case**	Parasitological confirmation of diagnosis by blood film or tissue histology. Cases treated presumptively or diagnosed by other methods (e.g. antigen based) are not included [34].	Indicated by diagnostic codes for malaria, or a combination of investigation and treatment codes
**Coverage and representativeness of sample**	UK population [2]	Coverage of around 6% of the UK population and has been shown to be broadly representative of the UK population [15]
**Data quality and type of data available**	Data collected solely for malaria surveillance capturing important variables related to malaria e.g. parasite species, ethnicity, travel history, chemoprophylaxis and treatment.	Primary use of EHR’s is patient management and data will reflect only those relevant to patient care. Good quality of recordings for investigations and prescriptions. Poor quality of data on parasite species, ethnicity and travel history.
**Timeliness of annual reporting**	Annual data is published six months after the end of the year (e.g. Annual report for 2016 available online August 2017)	Data is collected from participating general practices every three months by the data provider (IMS health/IQVIA), who then provide access to the data for researchers through a license.
**Strengths**	Most complete source of information about malaria in the UK Data is collected in a standardised way High specificity for identifying malaria cases	Does not rely on notification of cases. Availability of data on malaria investigation and treatment allows cases to be identified even when a diagnosis is not coded. Data is available on the sequence of care prior to a diagnosis of malaria, allowing investigation into potential missed opportunities for diagnosis and treatment. More accurate estimation of geographical distribution of cases Can explore associations for contracting malaria in variables not captured by the PHE malaria form
**Limitations**	Relies on notification—only 56% of cases captured by surveillance system Data available is restricted to what is collected in the Patient report/referral form Centralised reporting site—More sensitive to cases resident in London [6]	Miscoding, misclassification and misdiagnosis—These can be minimised by using recorded data from a consultation to exclude common coding errors (e.g. Miscoding malaria for malaria prophylaxis can be excluded by identifying prescriptions which are prescribed prior to travel) Does not capture those not registered with a GP Relies on practices using a specific IT system (INPS vision). Regional variation in the transition of practices to other IT systems can affect the representativeness of the sample over time.

This is the first study, to the best of our knowledge, which has explored malaria recordings over time using UK primary care data. The comparability of our findings to that from other UK data sources supports the use of this routinely collected data source for further research. Since the majority of previous malaria related research is retrospective [35], the routine recording in primary care datasets can allow prospective evaluation of the sequence of care prior to a diagnosis amongst the cases identified in this study. Given that prompt diagnosis and treatment of malaria remains a challenge in primary care [36], future research can use primary care data to identify missed opportunities for diagnosis and explore factors associated with it. Additionally, the improved geographic information on the distribution of cases can help with resource allocation and delivery of malaria prevention and treatment services.

## Conclusion

There has been a decrease in the number of malaria recordings in UK primary care over the past decade. This decrease exceeds the rate of decline reported in national surveillance data; however there are similar associations with age, sex and deprivation. Improved geographic information on the distribution of cases and the potential for automation of case identification suggests that EHRs could provide a complementary role for investigating malaria trends over time.

## Supporting information

S1 Fig Incidence of malaria recording by UK region in THIN. (DOCX)

S1 Table Read code lists used in this study. (DOCX)
