# Supplementary material for: Imported malaria in the UK, 2005 to 2016: Estimates from primary care electronic health records
Source: PLoS One. 2018 Dec 31;13(12):e0210040. doi: 10.1371/journal.pone.0210040 (PMC6312224; doi:10.1371/journal.pone.0210040)
Supplement: S1 Fig — (DOCX) [file pone.0210040.s001.docx]

**S1 Figure - Incidence of malaria recording by UK region in THIN**

**
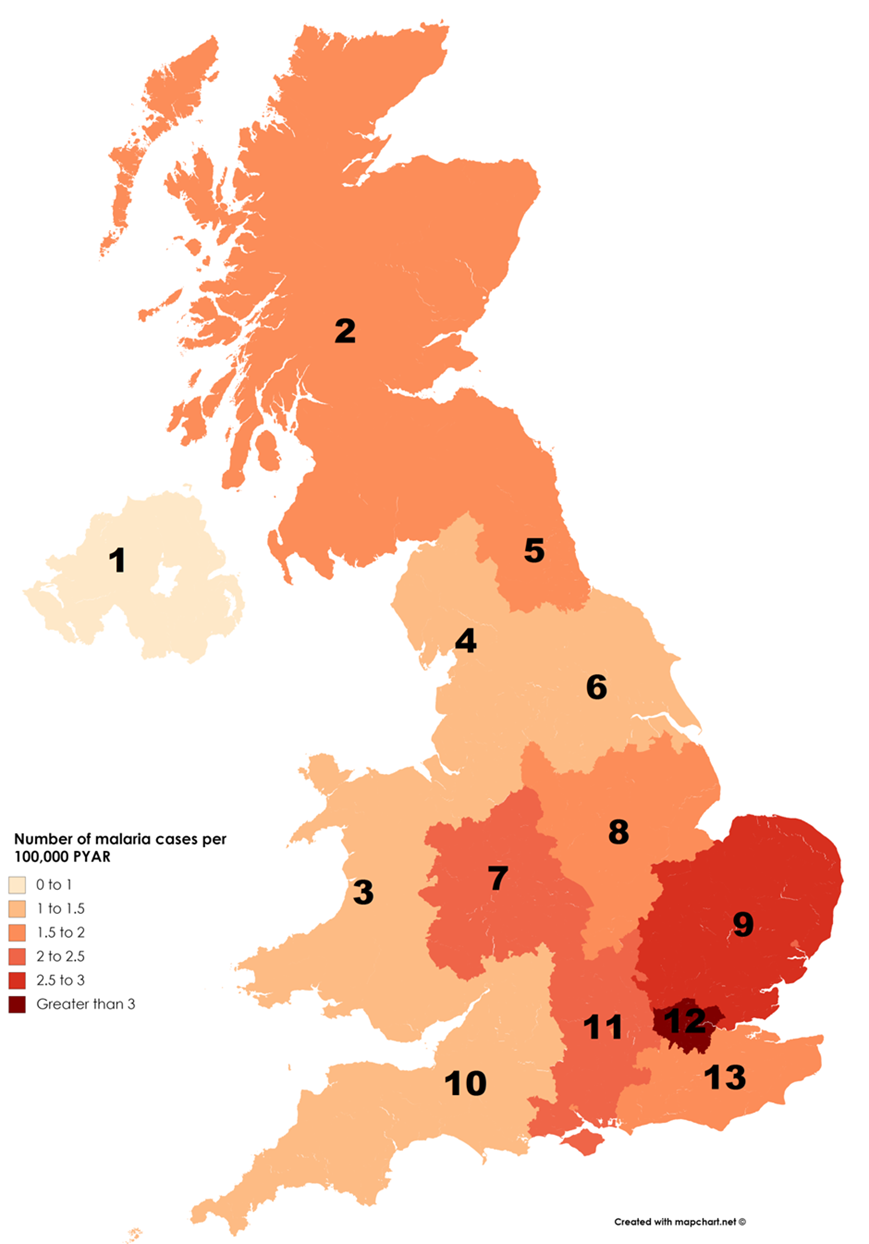
**

Rates of malaria recording per 100,000 PYAR by former Strategic Health Authority regions from 2005 to 2016. 1. Northern Ireland 2. Scotland 3. Wales 4. North West 5. North East 6. Yorkshire and the Humber 7. West Midlands 8. East Midlands 9. East of England 10. South West 11. South Central 12. London 13. South East Coast. Reprinted from mapchart.net under a CC BY license, with permission from Minas Giannekas original copyright 2018.
