# Supplementary material for: Imported malaria in the UK, 2005 to 2016: Estimates from primary care electronic health records
Source: PLoS One. 2018 Dec 31;13(12):e0210040. doi: 10.1371/journal.pone.0210040 (PMC6312224; doi:10.1371/journal.pone.0210040)
Supplement: S1 Table — (DOCX) [file pone.0210040.s002.docx]

**S1 Table**

Read codes used to identify malaria cases, malaria investigation and medications prescribed for malaria treatment.

## 1. Read codes used to identify cases of malaria

| Medcode | Description of malaria diagnostic Readcode |
| --- | --- |
| A84..00 | Malaria |
| A84X.00 | Plasmodium falciparum malaria, unspecified |
| A840.00 | Falciparum malaria - malignant |
| A846.00 | Unspecified malaria |
| A841.00 | Vivax malaria - benign tertian |
| A841X00 | Plasmodium vivax malaria without complication |
| A844.00 | Other malaria |
| A849100 | Cerebral malaria |
| 65V4.00 | Notification of malaria |
| A84W.00 | Plasmodium malariae malaria without complication |
| A843.00 | Ovale malaria |
| 42YA.00 | Malarial parasites |
| A840000 | Plasmodium falciparum malaria with cerebral complications |
| A849z00 | Other malaria complications NOS |
| F032011 | Malarial encephalitis |
| AyuF100 | [X]Plasmodium falciparum malaria, unspecified |
| J632000 | Hepatitis in malaria |
| A844.11 | Monkey malaria |
| A844000 | Malaria due to simian plasmodia |
| AyuF700 | [X]Unspecified malaria |
| F032000 | Encephalitis due to malaria |
| A845.00 | Mixed malaria |
| A844100 | Plasmodium malariae malaria with nephropathy |
| AyuF500 | [X]Plasmodium malariae malaria without complication |
| A847.00 | Induced malaria |
| A841000 | Plasmodium vivax malaria with rupture of spleen |
| A849.00 | Other malaria complications |
| K01x200 | Nephrotic syndrome in malaria |

## 2. Malaria specific investigation used in the algorithm to identify malaria cases

| Medcode | Description of malaria investigation |
| --- | --- |
| 6841 | Malaria screening |
| ZV75100 | [V]Screening for malaria |
| 4JP0.00 | Thick film for malarial parasites |
| 4JRD.00 | Malarial parasite screening test |
| 42Y1.00 | Microscopy for malarial parasites |
| 4JR6.00 | ICT malaria screening test |
| 4JDR.00 | Malaria serology |
| 4JQ5.00 | Malaria antigen test |
| AHD code |  |
| 1001400279 | Microscopy for malarial parasites |

## 3. Medications used to treat malaria and their corresponding Read codes in THIN

| Drugcode | Medication |
| --- | --- |
| 97987996 | Quinine sulfate 300mg tablets |
| 96305997 | Doxycycline 100mg capsules |
| 97987997 | Quinine sulfate 200mg tablets |
| 94959998 | Quinine bisulfate 300mg tablets |
| 88398998 | Proguanil 100mg / Atovaquone 250mg tablets |
| 92613998 | Doxycycline 50mg capsules |
| 98969998 | Doxycycline 100mg capsules |
| 97753998 | Doxycycline 50mg capsules |
| 96305996 | Doxycycline (as hyclate) 100mg tablets |
| 88399998 | Proguanil 100mg / Atovaquone 250mg tablets |
| 99291998 | Proguanil 100mg tablets |
| 95387998 | Proguanil 100mg tablets |
| 91630998 | Doxycycline 100mg dispersible tablets sugar free |
| 88368998 | Proguanil 100mg / Atovaquone 250mg tablets |
| 95532992 | Quinine bisulfate 300mg tablets |
| 88886998 | Proguanil 25mg / Atovaquone 62.5mg tablets |
| 99613990 | Doxycycline 100mg capsules |
| 93923998 | Doxycycline 100mg capsules |
| 96305998 | Doxycycline 100mg dispersible tablets sugar free |
| 94330992 | Quinine sulfate 300mg tablets |
| 99409990 | Quinine bisulfate 300mg tablets |
| 98969997 | Doxycycline 100mg dispersible tablets sugar free |
| 88271998 | Doxycycline 50mg capsules |
| 94328992 | Quinine sulfate 300mg tablets |
| 97246992 | Doxycycline 100mg capsules |
| 82732998 | Doxycycline 40mg modified-release capsules |
| 88230998 | Chloroquine phosphate 250mg tablets and Proguanil 100mg tablets |
| 97987998 | Quinine sulphate 125mg tablet |
| 98670989 | Quinine sulfate 300mg tablets |
| 93484992 | Doxycycline 100mg capsules |
| 88220998 | Chloroquine phosphate 250mg tablets and Proguanil 100mg tablets |
| 98231998 | Doxycycline 100mg dispersible tablets sugar free |
| 99451998 | Pyrimethamine & dapsone 12.5mg+100mg tablets |
| 99101998 | Doxycycline 100mg capsules |
| 88030998 | Doxycycline 50mg capsules |
| 96304997 | Doxycycline (as hyclate) 20mg capsules |
| 94534992 | Quinine bisulfate 300mg tablets |
| 88226998 | Chloroquine phosphate 250mg tablets and Proguanil 100mg tablets |
| 93614979 | Proguanil 100mg / Atovaquone 250mg tablets |
| 90801998 | Doxycycline 20mg tablets |
| 90963998 | Proguanil 25mg / Atovaquone 62.5mg tablets |
| 99405989 | Quinine sulfate 300mg tablets |
| 99613989 | Doxycycline 50mg capsules |
| 98601989 | Doxycycline 100mg capsules |
| 99101997 | Doxycycline 50mg capsules |
| 97761989 | Doxycycline 100mg capsules |
| 82730998 | Doxycycline 40mg modified-release capsules |
| 87995998 | Proguanil 25mg / Atovaquone 62.5mg tablets |
| 94536992 | Quinine sulfate 200mg tablets |
| 93642979 | Quinine bisulfate 300mg tablets |
| 98103990 | Quinine bisulfate 300mg tablets |
| 94961998 | Quinine dihydrochloride 300mg tablet |
| 94960998 | Quinine hydrochloride 300mg tablet |
| 95359998 | Pyrimethamine 25mg tablets |
| 99405990 | Quinine sulfate 200mg tablets |
| 98551998 | Pyrimethamine & sulfadoxine 25mg+500mg tablets |
| 99404990 | Quinine sulfate 300mg tablets |
| 95531992 | Quinine sulfate 200mg tablets |
| 97984992 | Quinine dihydrochloride 200 mg tab |
| 99404989 | Quinine sulfate 200mg tablets |
| 99410990 | Quinine bisulfate 300mg tablets |
| 98969996 | Doxycycline 50mg/5ml syrup |
| 98446990 | Quinine bisulfate 300mg tablets |
| 96304998 | Doxycycline (as hyclate) 50mg/5ml oral solution |
| 92856998 | Doxycycline 100mg capsules |
| 99402990 | Quinine sulfate 200mg tablets |
| 98697998 | Primaquine 7.5mg tablets |
| 99402989 | Quinine sulfate 300mg tablets |
| 92362998 | Doxycycline 100mg capsules |
| 89467998 | Doxycycline 20mg tablets |
| 97909990 | Quinine bisulfate 300mg tablets |
| 88431998 | Doxycycline 100mg capsules |
| 97209989 | Doxycycline 100mg capsules |
| 92521997 | Atovaquone 750mg/5ml oral suspension sugar free |
| 94662990 | Quinine sulfate 200mg tablets |
| 99403989 | Quinine sulfate 300mg tablets |
| 99078990 | Quinine bisulfate 300mg tablets |
| 95358998 | Dapsone 100mg / pyrimethamine 12.5mg tablets |
| 98670990 | Quinine sulfate 200mg tablets |
| 98447990 | Quinine bisulfate 300mg tablets |
| 98601990 | Doxycycline 50mg capsules |
| 99403990 | Quinine sulfate 200mg tablets |
| 99786998 | Pyrimethamine 25mg tablets |
| 99406990 | Quinine sulfate 300mg tablets |
| 95538992 | Quinine sulphate 500 mg tab |
| 96838990 | Quinine bisulfate 300mg tablets |
| 99408990 | Quinine bisulfate 300mg tablets |
| 94036992 | Quinine bisulphate 150 mg tab |
| 98044990 | Doxycycline 100mg capsules |
| 95357998 | Pyrimethamine 25mg / sulfadoxine 500mg tablets |
| 97913998 | Doxycycline 100mg tablets |
| 79282979 | Quinine sulfate 300mg/5ml oral suspension |
| 93613979 | Proguanil 100mg / Atovaquone 250mg tablets |
| 98352990 | Doxycycline 100mg capsules |
| 96354990 | Doxycycline 100mg capsules |
| 92613997 | Doxycycline 50mg capsules |
| 95533992 | Quinine bisulphate 125 mg tab |
| 95534992 | Quinine sulphate .5 gm tab |
| 95537992 | Quinine sulphate 250 mg tab |
| 97121990 | Doxycycline 100mg capsules |
| 95539992 | Quinine sulphate 100 mg tab |
| 92856997 | Doxycycline 50mg capsules |
| 79255978 | Proguanil 100mg / atovaquone 250mg tablets |
| 94331992 | Quinine sulphate 200 mg sus |
| 97051990 | Doxycycline 100mg capsules |
| 97209990 | Doxycycline 50mg capsules |
| 98352989 | Doxycycline 50mg capsules |
| 96282990 | Doxycycline (as hyclate) 100mg tablets |
| 92775990 | Doxycycline 100mg capsules |
| 96202990 | Doxycycline 50mg capsules |
| 96457998 | Dapsone 100mg with pyrimethamine 12.5mg tablets |
| 97921990 | Quinine sulfate 200mg tablets |
| 98444990 | Quinine sulfate 300mg tablets |
| 79250978 | Proguanil 25mg / atovaquone 62.5mg tablets |
| 79773978 | Proguanil 100mg / Atovaquone 250mg tablets |
| 82198978 | Proguanil 100mg / Atovaquone 250mg tablets |
| 92521998 | Atovaquone 250mg tablets |
| 93612979 | Proguanil 100mg / Atovaquone 250mg tablets |
| 94329992 | Quinine sulphate 4 mg tab |
| 96089990 | Doxycycline 100mg capsules |

## 4. Travel vaccinations and their corresponding Read codes in THIN

| AHD code | Description of travel vaccine |
| --- | --- |
| 1002000001 | Tetanus |
| 1002000010 | Polio |
| 1002000100 | Diphtheria |
| 1002020000 | Measles |
| 1002030000 | Mumps |
| 1002040200 | Rubella |
| 1002080000 | Hepatitis B |
| 1002080012 | Hepatitis A jr |
| 1002090000 | Influenza |
| 1002100000 | Pneumococcal |
| 1002100010 | Pneumococcal conjugate vaccine |
| 1002110000 | Rabies |
| 1002130000 | Typhoid |
| 1002132000 | Typhoid |
| 1002133000 | Typhoid |
| 1002150000 | Yellow Fever |
| 1002210003 | Meningococcal Polysaccharide ACWY |
| 1002340000 | Hepatitis A |
| 1002340005 | Hepatitis A |
| 1002344000 | Hepatitis A stage 2 |
